# Rabbits as a Reservoir of Multidrug-Resistant *Escherichia coli*: Clonal Lineages and Public Health Impact

**DOI:** 10.3390/antibiotics13040376

**Published:** 2024-04-20

**Authors:** Adriana Silva, Vanessa Silva, Teresa Tavares, María López, Beatriz Rojo-Bezares, José Eduardo Pereira, Virgílio Falco, Patrícia Valentão, Gilberto Igrejas, Yolanda Sáenz, Patrícia Poeta

**Affiliations:** 1 Microbiology and Antibiotic Resistance Team (MicroART), Department of Veterinary Sciences, University of Traás-os-Montes and Alto Douro (UTAD), 5000-801 Vila Real, Portugalteresatavares.vet@gmail.com (T.T.); 2 LAQV-REQUIMTE, Department of Chemistry, NOVA School of Science and Technology, Universidade Nova de Lisboa, 2829-516 Caparica, Portugal; 3 Department of Genetics and Biotechnology, University of Traás-os-Montes and Alto Douro (UTAD), 5000-801 Vila Real, Portugal; 4 Functional Genomics and Proteomics Unit, University of Traás-os-Montes and Alto Douro (UTAD), 5000-801 Vila Real, Portugal; 5 José Azevedo Monteiro, Lda., Rua do Campo Grande 309, 4625-679 Vila Boa do Bispo, Portugal; 6 Área de Microbiología Molecular, Centro de Investigación Biomédica de La Rioja (CIBIR), 26006 Logroño, Spain; mlopezm@riojasalud.es (M.L.); brojo@riojasalud.es (B.R.-B.);; 7 CECAV—Veterinary and Animal Research Centre, University of Trás-os-Montes and Alto Douro, 5000-801 Vila Real, Portugal; jeduardo@utad.pt; 8 Associate Laboratory for Animal and Veterinary Sciences (AL4AnimalS), 5000-801 Vila Real, Portugal; 9 Centre for the Research and Technology of Agro-Environmental and Biological Sciences (CITAB), Universidade de Trás-os-Montes e Alto Douro (UTAD), 5000-801 Vila Real, Portugal; 10 Laboratory for Green Chemistry (LAQV) of the Network of Chemistry and Technology (REQUIMTE), Universidade do Porto (UP), 2829-516 Caparica, Portugal; valentao@ff.up.pt; 11 REQUIMTE/LAQV, Laboratório de Farmacognosia, Departamento de Química, Faculdade de Farmácia, Universidade do Porto, 4050-313 Porto, Portugal

**Keywords:** *Escherichia coli*, antibiotic resistance, food safety, rabbit farms, multidrug resistance, animal production

## Abstract

*Escherichia coli*, including extended-spectrum β-lactamases (ESBL)-producing strains, poses a global health threat due to multidrug resistance, compromising food safety and environmental integrity. In industrial settings, rabbits raised for meat have the highest consumption of antimicrobial agents compared to other food-producing animals. The European Union is facing challenges in rabbit farming as rabbit consumption declines and antibiotic-resistant strains of *E. coli* cause enteric diseases. The aim of this study was to investigate the antibiotic resistance profile, genetic diversity, and biofilm formation in cefotaxime-resistant *E. coli* strains isolated from twenty rabbit farms in Northern Portugal to address the effect of the pressing issue of antibiotic resistance in the rabbit farming industry. Resistance to critically antibiotics was observed, with high levels of resistance to several categories, such as tetracycline, ampicillin, aztreonam, and streptomycin. However, all isolates were susceptible to cefoxitin and imipenem. Multidrug resistance was common, with strains showing resistance to all antibiotics tested. The *bla*CTX-M variants (*bla*CTX-3G and *bla*CTX-M9), followed by the tetracycline resistance genes, were the most frequent resistance genes found. ST10 clones exhibiting significant resistance to various categories of antibiotics and harboring different resistance genes were detected. ST457 and ST2325 were important sequence types due to their association with ESBL-*E. coli* isolates and have been widely distributed in a variety of environments and host species. The strains evaluated showed a high capacity for biofilm formation, which varied when they were grouped by the number of classes of antibiotics to which they showed resistance (i.e., seven different classes of antibiotics, six classes of antibiotics, and three/four/five classes of antibiotics). The One Health approach integrates efforts to combat antimicrobial resistance in rabbit farming through interdisciplinary collaboration of human, animal, and environmental health. Our findings are worrisome and raise concerns. The extensive usage of antibiotics in rabbit farming emphasizes the urgent need to establish active surveillance systems.

## 1. Introduction

*Escherichia coli* poses a serious threat to human and animal health and compromises food safety [1]. The emergence of multidrug resistance (MDR) has aggravated the situation with the emergence and dissemination of strains harboring extended-spectrum β-lactamases (ESBL) [2,3]. These β-lactamases contribute to antimicrobial multidrug resistance, since the strains that express these enzymes often show simultaneous resistance to other classes of antibiotics. In addition, the continuous mutations in the genes that encode β-lactamases, which are a direct response to the overuse of antibiotics, broaden their spectrum of action [4,5], and their continued mutation is a direct response to the overuse of antibiotics [6]. The global distribution of ESBLs and MDR *E. coli* represents a growing issue that extends beyond medical/healthcare systems and impacts food safety and environmental contamination [5]. ESBL-producing bacteria can be transmitted among humans and animals through the food chain and the environment [4].

Frequent use of antibiotics in livestock farming has been criticized for promoting antibiotic resistance. Factors that influence antibiotic resistance include farm management, water treatment, fertilizer handling, and wildlife management [2]. Contamination of food with antimicrobial-resistant bacteria occurs due to antibiotic use and through cross-contamination directly intended for human consumption [1,7]. The emergence of antimicrobial resistance poses a serious threat to human and animal health, and the widespread use of antibiotics in food animals has raised global concerns that impact both veterinary and human medicine. Many of the antibiotics used in humans are also being used in animal therapy [8]. Rabbits reared for meat in industrial farms exhibit the highest rates of antimicrobial usage compared to other food-producing animals, leading to alarming rates of antimicrobial resistance within the industry [9].

The European Union is the second largest producer of meat rabbits in the world, after China. The Union dominates global imports and exports, accounting for 93% of the market share, with Germany, Belgium, and Portugal being the main importers. Together, these countries contribute 14% of the rabbit meat in Europe; however, over the past two decades, the number of commercial rabbit farms has decreased across the European Union due to a decline in rabbit meat consumption [10]. *E. coli* is principally responsible for neonatal and post-weaning colibacillosis in rabbits, which is frequently accompanied by enteritis and diarrhea. The RESAPATH surveillance system produced susceptibility data for *E. coli* isolates from rabbits in 2020, with the majority coming from digestive pathology cases in 2018. Of the 277 isolates examined, 70.8% were associated with digestive problems, and treatment frequently included sulfonamides, fluoroquinolones, and aminoglycosides. Sulfonamides-trimethoprim had a greater resistance rate, although fluoroquinolone resistance was rare. Only 1% of isolates were resistant to third-generation cephalosporin [11]. Breeding rabbits is a health-risky industry, often suffering from high economic losses due to enteric diseases caused by *E. coli* colonization in commercial farms [12].

Antibiotic resistance in rabbits is a pressing issue due to limited research linking clonal *E. coli* strains to resistance, mainly in Portugal. High levels of resistance have been observed in Italian [13] and Chinese [14] studies, with consistent prevalence of β-lactamase genes like *bl*aTEM and *bla*CTX-M across studies [7]. Colistin resistance in rabbits is a concern in Portugal [13], as it is potentially transmitted through rabbit husbandry systems and the food chain. Common findings include increased resistance to major antibiotics and specific β-lactamase genes. Common sequence types like ST40 suggest the clonal and zoonotic potential of antibiotic-resistant *E. coli* strains [7].

Antibiotic resistance in commensal bacteria from food animals is a global concern, with research focusing on the effects of antibiotic use on animals and the potential transmission of resistant bacteria to humans. The use of antibiotics in food-producing animals has led to the development of MDR food bacteria like *E. coli*. While studies have primarily focused on *E. coli’s* prevalence in other livestock animals, there is a significant lack of research on rabbits [4]. Rabbit breeding, despite being a niche farm business, can lead to the spread of MDR bacteria, and in Portugal, where rabbit meat consumption is common, there have been no comprehensive studies analyzing a large number of farms to identify any consistent patterns in the results [15]. Therefore, the aim of our study was to investigate the prevalence of Cefotaxime (CTX)-resistant *E. coli* in healthy rabbits from 20 different intensive farms across northern Portugal, where there is a high concentration of rabbit farms. This work provides the distribution of antibiotic resistance profiles, virulence, genetic diversity, and biofilm formation of CTX-resistant *E. coli* populations in healthy rabbits from various farms across Portugal and highlights the potential public health implications that affects farmers, food, the environment, and surrounding crops. *E. coli* poses a significant threat to rabbit meat production and, consequently, food safety.

## 2. Results

### 2.1. Bacteria Isolation

From October 2022 to February 2023, a total of 295 fecal samples were received from 20 different rabbit farms. Cefotaxime (CTX)-resistant *E. coli* isolates were isolated from 48 samples, and these 48 isolates (16.27%) were obtained from 6 rabbit farms (Farm 2, Farm 3, Farm 4, Farm 5, Farm 6, and Farm 13).

### 2.2. Antibiotic Resistance Phenotypes

**Figure 1:**
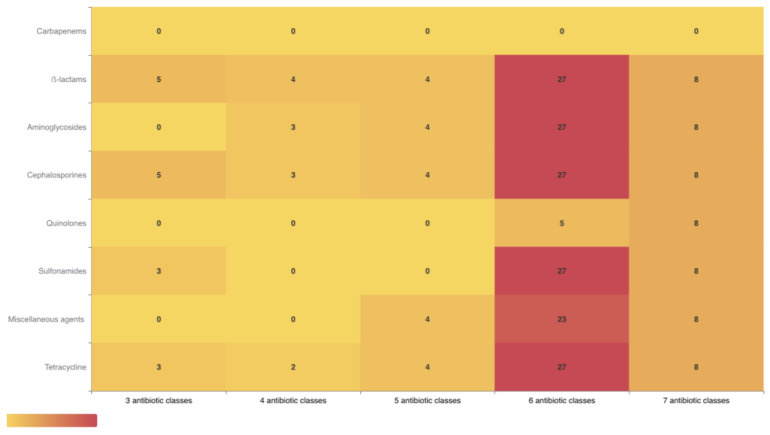
Heatmap showing the multiple resistance profiles of CTX-resistant *E. coli* isolates isolated from rabbit farms. The resistance to six different classes of antibiotics and seven different classes of antibiotics had highest association with the number of isolates in comparison to resistant to three, four and five different classes of antibiotics. The grading numbers in color strip depicts the number of different classes of antibiotics. The copy number, ranging from 0 to 27, was indicated by yellow to red.

The study examined the resistance patterns of various antibiotics in both critically important antimicrobials for the human therapeutic (CIA) and critically important veterinary antibiotics (VCIA) categories. It found ampicillin, amoxicillin-clavulanic acid, amikacin, gentamicin, streptomycin, tobramycin, and ciprofloxacin in both categories; ceftazidime, cefotaxime, aztreonam, and imipenem in CIA categories; and tetracycline in both categories. The 48 CTX-resistant *E. coli* isolates showed high rates of resistance to critically important antibiotics used in both human and veterinary medicine: ampicillin (100%), aztreonam (97.8%), and streptomycin (93.7%). Among the antibiotics tested, the following resistance rates were found: amoxicillin + clavulanic acid (54.16%), amikacin (8.3%), gentamicin (10.41%), tobramycin (64.58%), ceftazidime (14.58%), nalidixic acid and ciprofloxacin (25%), trimethoprim/sulfamethoxazole (75%), tetracycline (91.6%), and chloramphenicol (72.9%). The broad-spectrum β-lactam antibiotics cefoxitin and imipenem remain effective against all isolates. As Figure 1 shows, all 48 CTX-resistant *E. coli* isolates exhibited MDR profiles to multiple classes of antibiotics (at least three classes of antimicrobial agents). Eight isolates were resistant to seven different classes of antibiotics, and twenty-seven isolates were resistant to six different classes of antibiotics. Additionally, four isolates exhibited resistance to five classes of antibiotics, and six isolates exhibited resistance to three different classes of antibiotics.

### 2.3. Molecular Characterization and Multilocus Sequence Typing (MLST)

The ESBL-encoding genes *bla*CTX-M, *bla*TEM, *bla*SHV, and *bla*OXA were examined in all isolates according to the phenotypic resistance that they possess, and two different *bla*CTX-M variants were detected among our *E. coli* strains: *bla*CTX-3G (72.91%) and *bla*CTX-M9 (60.41%) (Table 1). The *bla*TEM was detected in 62.5% of our isolates, *bla*SHV in 6.25% of isolates, and *bla*OXA in none of the isolates. Several other resistance genes were also detected in our study, with lower prevalence. These genes included *tetA* (66.6%) and *tetB* (33.3%), associated with resistance to tetracycline; and *aac(6*′*)-Ib (*18.75%), *aac(3)-II* (10.41%), and *aac(3)-IV* (10.41%), associated with aminoglycoside resistance. The *qnr*A (20.83%) and *qnrS* (20.83%) genes, both associated with resistance to quinolones, were also identified. The *sul3* gene, associated with resistance to sulfonamides, was found in 64.58% of the isolates, *sul1 i*n 35.41%, and *sul2* in 29.16% of the CTX-resistant *E. coli* isolates. The *cmlA* gene, associated with chloramphenicol resistance, was detected in 43.75% of the isolates. Furthermore, the integrase gene (*intI1*) was found in 32 isolates, suggesting the presence of class 1 integrons and their involvement in rearrangement of gene cassettes and in the development of antibiotic resistance. Genes associated with virulence factors were widespread, and they were found in all *E. coli* isolates in this study, including *fimA*, *bfp*, *aer*, *cnf1*, *papC*, and *papG-II*. In Table 1 it is possible to observe the phenotypic and genotypic results of 29 strains of CTX-resistant *E. coli* that were not subjected to analysis by MLST. In the case of Table 2, we verified the phenotypic and genotypic analysis and sequence types of the 19 CTX-resistant *E. coli* isolates chosen for the MLST analysis.

**Figure 2:**
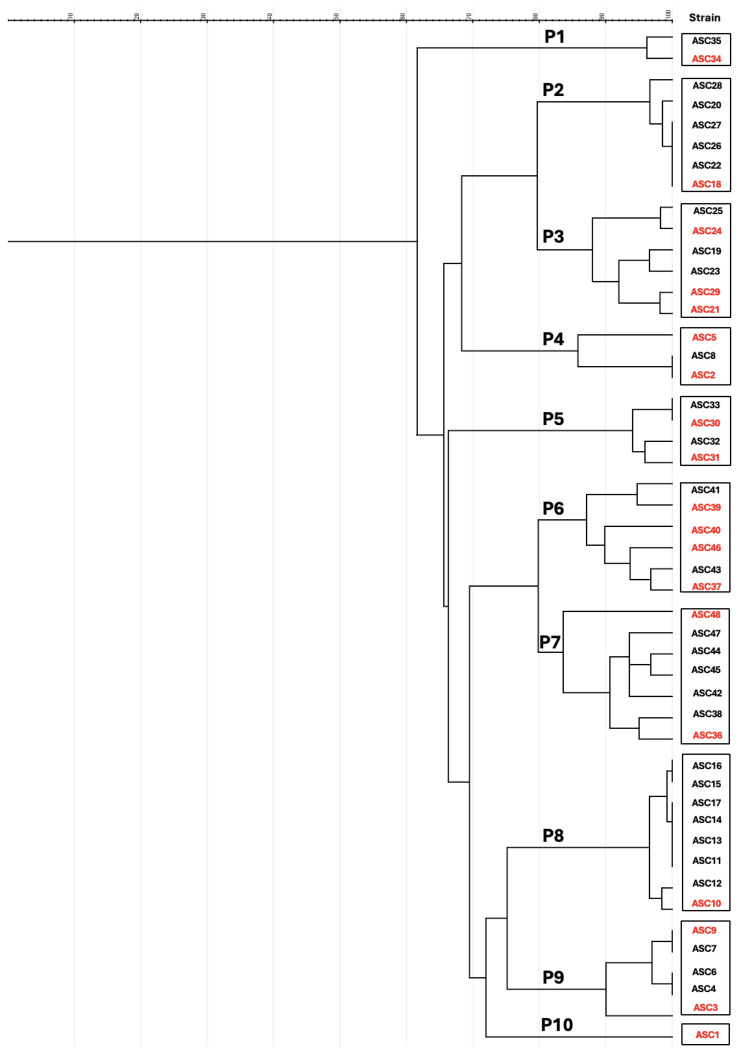
PFGE dendrogram of CTX-resistant *E. coli* strains from different rabbit farms in the north of Portugal. Braces indicate classification in the corresponding PFGE cluster or pulsotype. Isolates were included in the same pulsotype if their similarity indices were ≥80%. The strains selected to perform the MLST are highlighted in red.

Phylogenetic group A was the most prevalent (56.25%), followed by group B1 (37.5%) and group D (2.08%). The 48 isolates analyzed belonged to 10 different pulsotypes (Figure 2). The sequence types (ST) were determined among 19 *E. coli* strains (at least one strain per PFGE pattern) using the MLST method. Seven different ST were observed: ST10, ST457, ST1611, ST2325, ST2825, ST8470, and ST8823.

### 2.4. Quantification of Biofilm Formation

**Figure 3:**
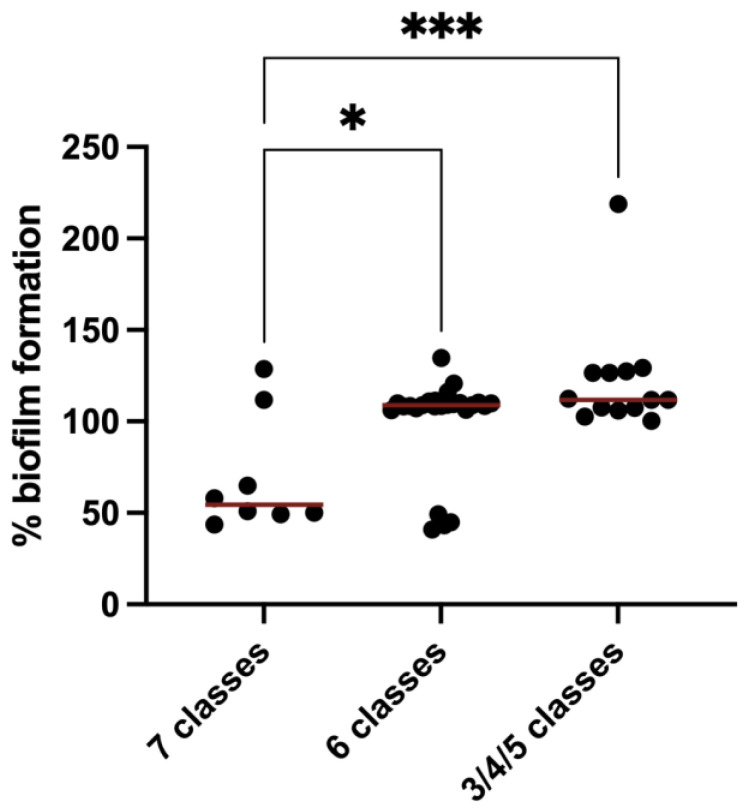
% Biofilm formation capacity (expressed as % in comparison to reference strain) of *E. coli* strains isolated from different rabbit farms. The strains were divided according to their resistance phenotypes. The symbols represent the biomass mean of the biofilm formed in independent tests of the individual isolates. The red lines represent the mean biofilm mass formed per group. Statistical significance was determined using Tukey’s multiple comparisons test (* *p* < 0.05; *** *p* < 0.001).

A microtiter plate assay was used to measure the biofilm production in all 48 CTX-resistant *E. coli* strains isolated. All isolates showed biofilm formation. To ensure consistency, the results were normalized against *E. coli* ATCC 25922. Figure 3 shows the biofilm formation of each isolate grouped by resistance phenotype (seven different classes of antibiotics, six classes of antibiotics and three/four/five classes of antibiotics). Strains belonging to three/four/five classes of antibiotics had a significantly higher average biofilm formation rate (*p* < 0.001) and showed the highest biofilm production, followed by those belonging to six classes of antibiotics (*p* < 0.05) and those belonging to seven classes of antibiotics (*p* < 0.001). The strain with the highest biofilm formation belonged to the group resistant to three/four/five classes of antibiotics, and the strain that produced the least biofilm mass belonged to the group resistant to 7 classes of antibiotics.

## 3. Discussion

The industrial production of rabbits for meat, despite being limited to a few countries, is unsustainable due to the global threat of antimicrobial resistance, high antibiotic levels, and potential sharing of MDR genotypes between farm workers and rabbits [16]. Oral medication, commonly administered to rabbits, potentially leads to under-dosing of large groups of animals in a herd and contributes to antimicrobial resistance development. Economic factors, particularly the costs associated with antimicrobial treatment, play a significant role, especially in less profitable livestock productions like rabbit meat [17]. Compared to other food-producing animals, rabbits raised for meat in industrial settings are the most abundant consumers of antimicrobial agents [16]. The study of *E. coli* antimicrobial resistance provides insights into the reservoir of resistant bacteria in healthy animals and their food, potentially facilitating the transfer of resistance between animal populations and humans [18].

### 3.1. Antibiotic Resistance in Rabbit Farm Environments

In our study, we performed an analysis of rabbit fecal samples, obtaining 15 samples per farm from 20 different rabbit farms located in the Trás-os-Montes, Alto Tâmega, and Minho regions. Among the 20 farms investigated, 48 CTX-resistant *E. coli* strains were isolated in samples from only six different farms (Farm 2, Farm 3, Farm 4, Farm 5, Farm 6 and Farm 13). None of these farms were related to each other, nor were they located in the same proximity. Additionally, we found that the positive samples (16.27%) exhibiting growth were detected in the Ave and Cávado regions, with no detection of CTX-resistant *E. coli* strains in the Trás-os-Montes, Alto Tâmega, or Douro regions. The locations where the samples tested positive for *E. coli* CTX-resistance were in closely populated areas, particularly in the immediate neighborhoods of major cities such as Braga, Barcelos, Vila Nova de Famalicão, and Guimarães. Additionally, three of these farms were located near livestock industries, including cattle and poultry farms. In contrast, all other farms where CTX-resistant *E. coli* strains were not detected were in remote areas, far from human settlements and residential areas. This represents a compelling example of the One Health approach and underscores the need to be vigilant of this pathogen in order to reduce the potential for zoonotic transmission and disease outbreaks. Several studies [19] have shown that *E. coli* isolated from rabbits can be considered a potential zoonotic transmission in pet rabbits, farm rabbits, and wild rabbits. Rabbits can serve as reservoirs for antimicrobial resistance genes, potentially spreading them to surrounding ecosystems, and may spread pathogenic bacteria in the environment [19]. Regarding domestic rabbits and pet rabbits, the dissemination of resistant bacteria can occur in different ways. In pet species, the close human–animal interface poses a potential public health risk for the transmission of zoonotic diseases from rabbits to their owners, particularly when good hygiene practices are not followed [20]. In meat rabbits, transmission can occur from rabbit to human and from rabbit to other animal through bacterial transmission (and vice versa) if biosecurity practices are poor. Regardless of farm size, the risk of disease transfer is significantly increased not just between rabbits, but also between rabbits and humans [21]. Regarding the results shown in our study, we found that farms located in urban areas have CTX-resistant *E. coli*, which could happen due to the zoonotic potential that *E. coli* has and due to the contamination, that can occur through soil, air, and water. Direct contact with environments contributes to contamination with antibiotic-resistant bacteria [22] The One Health approach emphasizes understanding the connections between human, animal, and environmental microbiota. Transmission of antibiotic resistance genes between livestock and humans can occur through direct and indirect contact. Soil and airborne transmission are also concerns [23].

The study examined seven antibiotics listed as both critically important antimicrobials for human therapeutics (CIA) and critically important veterinary antibiotics (VCIA), including ampicillin, amoxicillin–clavulanic acid, amikacin, gentamicin, streptomycin, tobramycin, and ciprofloxacin. Four were exclusively in the CIA category: ceftazidime, cefotaxime, aztreonam, and imipenem. Additionally, a few antibiotics, such as cefoxitin, chloramphenicol, and -trimethoprim/sulfamethoxazole, were included in the highly important antibiotics (HIA) list by the World Health Organization (WHO). Furthermore, tetracycline was listed in both the HIA and VCIA categories [24]. Our findings reveal alarming levels of resistance to several critically important antibiotics used in both human and veterinary medicine: tetracycline (91.6%), ampicillin (100%), aztreonam (97.8%), streptomycin (93.7%), tobramycin (64.58%), trimethoprim/sulfamethoxazole (75%), amoxicillin–clavulanic acid (54.16%), and chloramphenicol (72.9%). Nevertheless, some broad-spectrum β-lactam antibiotics (cefoxitin and ceftazidime), as well as nalidixic acid, remain effective, with low rates of phenotypic resistance (25%), as well as imipenem, for which no resistance was observed. The β-lactams are rarely used in rabbits due to drug-related diarrhea, leading to *E. coli* isolates being almost wild-type for CTX [16]. However, in this study, we found that antibiotics belonging to this class of antibiotics have high levels of resistance. Despite chloramphenicol having been banned in food-producing animals for 20 years, nearly one in four *E. coli* indicators showed reduced susceptibility to this drug, and occurrence has also been reported in other food-producing animals [16].

In our study, high resistance to this antibiotic was reported in our isolates. Limited studies have evaluated antibiotic resistance in domestic rabbits, but existing research suggests higher levels of resistance to tetracycline and ampicillin in other rabbit farms. A study in China [14] found increased levels of resistance to tetracycline and ampicillin (78.2% and 65.5%, respectively). However, our study found even higher levels of resistance compared to the study in China. Another study in Tunisia [12] reported similarly high rates of antibiotic resistance to tetracyclines, with the highest resistance being 95%. However, ampicillin showed the highest resistance rate in our study. High levels of tetracycline resistance have also been reported in rabbit farms worldwide due to the widespread use of tetracyclines for controlling and preventing rabbit diseases. The frequent use in both the veterinary and human health sectors has been cited as a contributing factor to the emergence and spread of tetracycline-resistant bacteria [25]. While our research focused on intensive rabbit farms, similar studies on wild rabbits in Europe have found resistance to *E. coli*. A study in northern Portugal [26] found that 57.1% of the samples tested positive for *E. coli* isolates with resistance patterns to antibiotics such as ampicillin, sulfamethoxazole/trimethoprim, and tetracycline. This resistance differs from higher levels reported in food-producing animals. Another study in wild rabbits in Azores [27] found *E. coli* isolates resistant to common antibiotics, suggesting that wild rabbits act as reservoirs of antimicrobial-resistant genes, similarly to those used for consumption. The presence of MDR strains poses a potential threat to public health, and the use of antibiotics in livestock production leads to the development of MDR and ESBL *E. coli* strains, making them difficult to treat, as well as a significant reservoir of resistance genes [7,14]. In our study, all *E. coli* isolates showed MDR, with eight isolates being resistant to seven different antibiotics classes, twenty-seven to six different classes, four to five different classes, and six to three different classes. The detection of MDR strains on all farms highlights a growing concern about rabbits as production animals. MDR pathogens pose a significant threat because they can cause severe and long-lasting infections, raising the possibility of a global pandemic [28]. Studies worldwide consistently show high rates of MDR among *E. coli* isolates, which often exhibit resistance to various antibiotics, indicating broad-spectrum antimicrobial agents [7]. This study examined antibiotic resistance genes in CTX-resistant *E. coli* isolates, analyzing β-lactamase genes and non-β-lactams resistance. The study found two groups of *bla*CTX-M in *E. coli*, *bla*CTX-3G (72.91%) and *bla*CTX-M9 (60.41%), among ESBL-producing *E. coli*. The emergence of ESBL-producing *E. coli* in food-producing animals is a major concern due to reduced treatment efficacy and increased morbidity and mortality rates. Studies have confirmed the presence of these bacteria in livestock, highlighting the need for a One Health strategy to combat antibiotic resistance [7]. Tetracycline resistance in *E. coli* isolates is facilitated by active efflux from tet*A* and tet*B* genes [27], which, in our study, had high rates of resistance. Aminoglycoside resistance genes (*aac(6)-*Ib, *aph(3)*, *acc(3)-*II, and *acc(3)-*IV), quinolone resistance genes (*qnr*A *and qnr*S), sulfonamide resistance genes (*sul2* and *sul3*), and chloramphenicol resistance genes (*cml*A) exhibited a significant prevalence among isolates resistant to the antibiotics for which they provide resistance. *bla*TEM is a β-lactamase gene and the primary cause of ampicillin resistance in *E. coli*. It is found in food sources, humans, and healthy animals in Spain [14]. In this study, 62.5% of *E. coli* isolates carried this gene. Previous studies of rabbit farming have found a similar prevalence of resistance genes to our study, as demonstrated by studies in Italy [13], China [14], and Portugal [29]. These studies reported a significant presence of genes conferring resistance to β-lactams, such as *bla*TEM and *bla*CTX, as well as genes related to tetracyclines, sulfonamides, and aminoglycosides. The study conducted in Portugal identified the *mcr-1* gene [22]; the presence of this gene was not detected in any *E. coli* isolates in our study. The rise in antibiotic resistance in *E. coli* isolates from intensive rabbit farms is consistent with similar trends in other animals used for consumption, such as swine, poultry, and cattle. Factors contributing to this resistance include population growth and increased meat production globally. Although antibiotics were banned for growth promotion in food-producing animals in 2006 to combat resistance and eliminate residues in meat, the industry continues to be a significant contributor to antibiotic resistance. Livestock, particularly poultry, swine, and dairy cattle, account for 50–80% of antibiotic use, resulting in high resistance to antibiotics like tetracyclines, sulfonamides, and penicillins [7]. This research suggests that the high levels of resistance in intensive rabbit farms reflect an overall trend of antibiotic resistance in various food-producing animals worldwide.

### 3.2. Genetic Diversity of CTX-Resistant E. coli in Rabbit Farms

The study analyzed 48 isolates from 10 different pulsotypes, and some isolates were clonally related. The study found that Farm 2 displayed the P10 cluster and Farm 3 had the highest clonal diversity among the farms, with eight isolates distributed across three distinct clusters (P1, P4, and P9). Farm 4 and Farm 6 each had a single cluster associated, the P8 and P5 clusters, respectively, while Farm 5 had 12 isolates divided into 2 distinct clusters (P2 and P3), and Farm 13 had isolates divided into two distinct clusters (P6 and P7). In all cases, similarities existed only between CTX-resistant *E. coli* strains isolated within each farm, and not between CTX-resistant *E. coli* strains from different farms. MLST analysis and clonal lineages were assessed using at least one strain per PFGE pattern. Seven different STs were observed among 19 *E. coli* strains: ST10, ST457, ST1611, ST2325, ST2825, ST8470, and ST8823. The study found high genetic diversity in six rabbit farms. Farm 2 and Farm 4 had the same clonal lineage, ST10, but the strains belonged to different pulsotypes. For Farm 2, the ST10 clone showed remarkable antibiotic resistance across seven classes and several resistance genes, indicating a significant genetic diversity in these farms. Phylogenetically, it fit into group A and contained virulence-associated genes like *fimA* and *bfp.* The other ST10 clone exhibited resistance to six different classes of antibiotics, but unlike the clone belonging to Farm 2, it did not exhibit resistance to the quinolones. Phylogenetically, it fit into group A and contained virulence-associated genes such as *papG*-III, *fimA,* and *bfp.* The identification of MDR *E. coli* ST10 clones as persistent One Health clones underscores the interconnection between humans, animals, and environmental health and has been documented across multiple hosts and sources, including high-risk pandemic lineages. Infections caused by high-risk strains are frequently resistant to most commercially available antibiotics, including antibiotics used as the last resort. These results emphasize the need for coordinated efforts to mitigate the spread of antimicrobial resistance strains across different sectors [30]. Regarding Farm 3, we selected four strains for analysis and confirmed the presence of two different STs, two strains of ST1611 (P4) and two strains of ST8470 (P9), which phylogenetically belong to B1 and A, respectively. These isolates have very complex resistance profiles: resistance to six and seven different classes of antibiotics, respectively. ST1611 has been reported in several livestock and food product studies in Italy [31], Poland [32], and China [33]. One of the studies involved rabbits in Italy [13]. Regarding ST8470, it is important to highlight that it belongs to clonal complex 10, such as ST10. According to Enterobase, it was detected in 2014 in a sample of humans in Denmark, and there is no further record of this ST in any other environment. This confirms its ubiquitous distribution between different reservoirs and the clonal cross-species transmission of *E. coli* in livestock animals and humans. On Farm 5, four different isolates were analyzed, all of which detected the ST2825 clonal strain, but the strains belonged to different pulsotypes (P2/P3). Studies conducted in livestock have found no evidence of this clonal lineage. However, this ST was first detected in marine sediments along the Adriatic coast, where MDR *E. coli* strains were isolated [34]. Regarding the isolates analyzed on Farm 6, we confirmed the presence of clonal lineage ST8823 in the two selected isolates. EnteroBase records demonstrate the presence of this ST in wildlife, environment, and poultry research geographically in Gambia, the United Kingdom, Kenya, the United States, and the United Arab Emirates from 2019 to 2013. Since this ST was detected only in poultry in 2023, we can conclude that this is the case, and since this ST was also found in rabbit breeding, is already widespread in livestock (https://enterobase.warwick.ac.uk (accessed on 6 February 2024)). Farm 13 was the last rabbit farm where CTX-resistant *E. coli* was isolated, with seven strains analyzed for MLST. Two ST types were detected, ST457 and ST2325, with ST2325 being the most common. The ST457 has been described as an emerging extraintestinal pathogenic *E. coli* mainly found in wildlife and in food-producing animals [35]. It has a wide host range with global distribution, indicating that ST457 has been reported from different sources in studies on all continents [35]; it is found in marine environments and bloodstream infections, and has been reported in healthy and sick humans, poultry, cattle, swine, wild animals, livestock, companion animals, water, and food [35,36,37]. This *E. coli* sequence, type ST457, showed a remarkable ability to capture mobile elements that carry and transmit genes encoding resistance to clinically important antibiotics [35]. ST2325 is the most widespread in our study, and has so far been detected in a variety of environments, including livestock [38], stray dogs [39], food products (meat), and the environment (soil) [40]. It is primarily associated with ESBL-*E. coli* isolates.

The identification of distinct STs, including high-risk and pandemic clones, highlights the potential public health implications of antimicrobial resistance in agricultural settings. It also emphasizes the interconnectedness of human, animal, and environmental health, as evidenced by the widespread distribution of certain STs across multiple reservoirs. The detection of the same STs in different environments and host species demonstrates their ability to adapt and spread widely, posing a significant threat to global health security.

### 3.3. Biofilm Formation in MDR E. coli Isolated from Rabbit Farms

*E. coli* biofilm production ability may pose a significant threat to food processing and production, as it increases bacterial resistance to disinfectants, increasing the risk of cross-infection and causing harm to consumer health [41]. In our study, we analyzed the biofilm formation of 48 CTX-resistant *E. coli*-grouped antibiotic resistance phenotypes. Strains showing resistance to three/four/five different categories had higher average biofilm formation rates and the highest biofilm production, followed by those in the six and seven classes. Several studies have aimed to determine the relationship between MDR profiles and their ability to form biofilms. In our study, all the strains were MDR, and we tried to relate the number of classes they would be resistant to with their ability to form biofilms. The results showed that the number of classes of resistance did not affect biofilm production, since resistance to seven classes was the category that resulted in lower biofilm production when compared to the others. Regarding other studies, there is no information concerning biofilm formation in *E. coli* strains isolated from rabbit farms. However, our results can be compared with other studies conducted on livestock and food products. A study in China found that 25.39% of *E. coli* strains from poultry meat formed biofilms, with high-producing strains found in beef [42]. Stronger biofilm-forming strains were also found in poultry isolates [43]. In Bulgaria, isolates from three industrial farms formed strongly adherent biofilms, indicating the presence of *E. coli* in various meat products [44]. The presence of resistance to commonly used antimicrobials, coupled with the occurrence of MDR strains and strong biofilm formation ability, is alarming. However, in our study, we were unable to verify that the number of antibiotic classes to which the strains are resistant influences biofilm formation. The antimicrobial treatment of biofilms leads the formation of persister cells that can tolerate high levels of antibacterial compounds, which continue to form biofilms even after treatment has finished, transmitting between reservoirs and infecting humans [45].

The rational use of and reduction in antibiotics in rabbit farms is hindered by antibiotic administration laws, economic constraints, a lack of biosafety standards, consumer demand, and inadequate training. The rabbit industry lacks biosecurity measures, leaving rabbits vulnerable to health risks. MDR strains are disseminated and classified as pandemic and high-risk clones, making them more susceptible to infections that could lead to mortality. There is growing demand, but less pressure from consumers, for rabbit meat products to meet specific standards to reduce antibiotic use [9]. The European Food Safety Authority (EFSA) emphasizes the need for measures to reduce antimicrobial use, including improving farmers’ understanding of herd hygiene and promoting different attitudes towards herd health management in different livestock sectors [17].

## 4. Materials and Methods

### 4.1. Sample Collection, Isolation, and Identification of Escherichia coli Isolates

**Figure 4:**
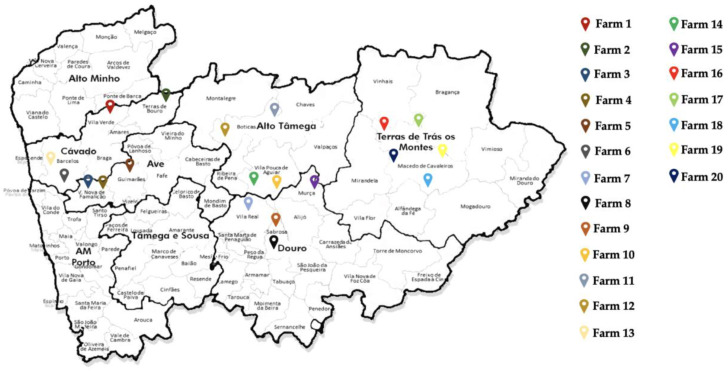
Geographic locations of rabbit farms. Samples were collected from 20 rabbit farms in the Trás-os-Montes, Alto Tâmega, Douro, Ave, and Minho regions. Each farm is marked in a different color.

A total of 295 fecal samples were collected from 20 rabbit farms in the Trás-os-Montes, Alto Tâmega, Douro, Ave and Minho regions between October 2022 and February 2023 (Figure 4). At each farm, 15 samples were collected from different locations in the rabbit hutch area to ensure significant samples and to cover the entire rabbit farm.

From each fecal sample, a 5 g aliquot was homogenized and diluted in brain heart infusion (BHI) broth and incubated at 37 °C for 24 h under aerobic conditions. After incubation, samples were plated on Chromocult^®^ Coliform Agar (ChromoCult, Fontenay sous Bois, France) supplemented with 2 µg/mL of cefotaxime. The plates were then incubated at 37 °C for 24 h. One colony per sample with the morphological aspect of *E. coli* was selected and inoculated onto eosin–methylene blue agar (EMB) and MacConkey agar plates at 37 °C for 24 h. Colonies presumed to have morphological characteristics consistent with *E. coli* were collected (1 colony per sample) and subjected to standard biochemistry including IMViC reactions (indole, methyl red, Voges–Proskauer, and citric acid). Matrix-assisted laser desorption/ionization time-of-flight mass spectrometry (MALDI-TOF MS, MALDI Biotyper^®^, Bruker Daltonik, Bremen, Germany) was used to confirm the species-level identification of bacterial isolates. The isolated *E. coli* strains were stored at −80 °C for subsequent characterization.

### 4.2. Antimicrobial Susceptibility Testing

The Kirby–Bauer disk diffusion method was used to assess antibiotic susceptibility on Mueller–Hinton (MH) agar according to the European Committee for Antimicrobial Susceptibility Testing (EUCAST) guidelines (2022) [46]. A total of 16 antibiotics (μg/disc), categorized as human therapeutic (CIA) and critically important veterinary antibiotics (VCIA), were tested for their relevance to human and animal health: ampicillin (10 μg), amoxicillin–clavulanic acid (AMC) (20 + 10 μg), cefoxitin (30 μg), ceftazidime (30 µg), aztreonam (30 µg), imipenem (10 µg), gentamicin (10 µg), amikacin (30 µg), tobramycin (10 µg), streptomycin (10 µg), nalidixic acid (30 µg), ciprofloxacin (5 µg), trimethoprim/sulfamethoxazole (SXT) (1.25/23.75 μg), tetracycline (30 μg), and chloramphenicol (30 μg). Incubation was carried out aerobically at 37 °C for 24 h. The classification of isolates as susceptible or resistant was determined based on the diameter of the zone of inhibition around the antibiotic disk according to the EUCAST breakpoints [46]. One isolate per stool sample was selected for further investigation.

### 4.3. Characterization of Antimicrobial Resistance Genes and Virulence Genotyping

Genomic DNA from *E. coli* isolates was extracted using the boiling method, and the presence of antibiotic resistance genes in cefotaxime-resistant isolates was investigated. The presence of b-lactamase genes was analyzed by PCR: *bla*CTX-M of different groups (groups 3 and 9), *bla*TEM, *bla*SHV, *and bla*OXA. Additionally, PCR was used to identify genes associated with non-beta-lactam resistance, including tetracyclines (*tet*A, *tet*M, and *tet*B), sulfonamides (*sul1*, *sul2* and *sul3*), streptomycin (*str*A and *str*B), chloramphenicol (*cml*A), aminoglycosides (*ant(2)*, *aph(3)*, *aac(3)-II*, *aac(3)-IV*, *aad*A1 and *aac(6*′*)-*Ib), quinolones (*qnr*S and *qnr*A), and colistin (*mcr-1*). The presence of *intI1* and *intI2* genes encoding class 1 and 2 integrases was also tested using PCR [47].

PCR assays were used to identify genes associated with virulence factors in *E. coli* isolates, including *fimA* (type 1 fimbriae), *papGIII* (adhesin PapG class III), *hlyA* (hemolysin), *cnf1* (cytotoxic necrotizing factor), *papC* (P fimbriae), *aer* (aerobactin iron uptake system), *eae* (Intimin), and *bfp* (Type IV bundle forming pili) [47].

### 4.4. Phylogenetic Diversity and Clonal Relationship

The identification of major phylogenetic groups (A, B1, B2, or D) among the *E. coli* isolates was established using PCR, incorporating a set of three genes (*chuA*, *yjaA*, and *TspE4.C2*), as outlined by Clermont et al. [48].

The clonal relationship between the different isolates was studied by pulsed-field gel electrophoresis (PFGE) using *XbaI* enzyme to digest genomic DNA, as previously reported [49]. The PFGE conditions were 6 V cm^−2^, 14 °C, and pulse time ranging from 1 s to 30 s over the course of 19 h using the CHEF-DR III system (Bio-Rad Laboratories Inc., Hercules, CA, USA). PFGE patterns were analyzed using the Java program GelJ v2 using the Dice coefficient [50]. Isolates with ≥80% of identity were classified as belonging to the same epidemiological clonal group [51]. At least one isolate per PFGE pattern was typed using multilocus sequence typing (MLST) with the Achtman scheme, involving PCR amplification of seven housekeeping genes. Subsequently, all amplicons were sequenced and compared against MLST database sequences to identify specific allele combinations and determine the sequence type (ST) [52].

### 4.5. Biofilm Formation

The biofilm formation assay was performed according to a previously outlined protocol [23], with some adjustments. Briefly, two fresh colonies from a culture were transferred into tubes containing 3 mL of tryptic soy broth (TSB, Oxoid, Basingstoke, UK) and incubated at 37 °C for 16 ± 1 h with continuous shaking at 120 rpm using a shaker incubator (ES-80 Shaker, Grant Instruments, Cambridge, UK). After this incubation, the bacterial suspension was standardized to an optical density equivalent to 1 × 10^6^ colony-forming units (CFUs), and then 200 μL of each isolate was added to individual wells of a 96-well flat-bottom microplate. *E. coli* ATTC 25922 served as a positive control and fresh, sterile medium as a negative control. The plates were incubated at 37 °C for 24 h without shaking, with seven technical replicates prepared for each experiment. Biofilm mass was evaluated using the crystal violet (CV) staining method, following the procedure described by Peeters et al. (2008) [53] with some modifications. After incubation, each well was washed twice with 200 μL of distilled water to remove non-adherent bacterial cells. The plates were air-dried at room temperature for approximately 2 h, and then 100 μL of methanol (VWR International, Carnaxide, Portugal) was added to fix the microbial biofilm, then allowed to react for 15 min. Subsequently, methanol was removed, and the plates were air-dried for 10 min at room temperature. Following this, 100 μL of 1% (*v*/*v*) CV solution was added to each well and allowed to sit for 10 min. Excess CV solution was removed by washing the plates twice with distilled water. To dissolve CV, 100 μL of 33% (*v*/*v*) acetic acid was added, and absorbance was measured at 570 nm using a microplate reader (Bio Tek elX808U, Winooski, VT, USA) [54]. Biofilm formation results for each isolate were presented as a percentage of the results obtained for the reference strain.

## 5. Conclusions

The emergence of CTX-resistant *E. coli* recovered from healthy rabbits from 20 different intensive farms across north Portugal highlights a concerning prevalence of antibiotic resistance in rabbit-farming environments and the spread of MDR bacteria. The increase in resistance to antibiotics frequently used in veterinary and human healthcare settings, such as tetracycline, ampicillin, aztreonam, and streptomycin, underscores the growing threat to public health and poses a significant challenge to One Health. The detection of MDR strains on all farms, including the identification of various high-risk and pandemic clones (ST10, ST457, and ST2325), coupled with their great ability to form biofilms, poses a substantial threat to food safety and consumer health and reflects the broad host adaptability and wide geographical spread of these organisms, which suggests the possibility of cross-species transmission and widespread distribution of genes conferring antimicrobial resistance. This study underscores the need for One Health strategies to address antimicrobial resistance in rabbit farming. Regulatory measures and consumer awareness campaigns are crucial for promoting sustainable practices and reducing antibiotic use, and, with these strategies, will ensure long-term sustainability, adaptability, public health, and environmental well-being in rabbit farming.

## Figures and Tables

**Table 1:** Phenotypic and genotypic characterization of CTX-resistant *E. coli* isolates that were not chosen for MLST analysis.

Isolate	Farm	Resistance Phenotype	Resistance Genotype	Phylogenetic Group	Integrase Gene	Virulence Genes	PFGE Pattern
ASC4	3	AMP-S-TOB-CTX-NA-CIP-SXT-C-TE	*sul1-sul3-qnrS- -strA-strB- bla*CTX-3G*-tetB-aac(6)-Ib*	A	*int1*	*fimA-bfp*	P9
ASC6	3	ATM-AMP-S-TOB-CTX-NA-CIP-SXT-C-TE	*sul1-sul3-tetA-qnrS-qnrA-—strA-strB- bla*CTX-3G*-tetB-aac(6)-Ib-bla*TEM	A	*int1*	*papG-III-fimA*	P9
ASC7	3	ATM-AMP-AK-CN-S-TOB-CTX-NA-CIP-SXT-C-TE	*sul1-sul3-tetA-tetB-qnrS-qnrA- -aac(3)-IV-aac(3)-II- strA- bla*CTX-3G*- aac(6)-Ib-bla*TEM	A	*int1*	*papG-III-fimA-bfp*	P9
ASC8	3	AUG-ATM-AMP-S-TOB-CTX-SXT-C-TE	*sul1-sul3-tetA -strA- strB-bla*CTX-3G	B1	*int1*	*fimA*	P4
ASC11	4	AUG-ATM-AMP-S-CTX-CAZ-SXT-C-TE	*sul2-sul3-tetA- strA-strB- bla*CTX-3G*-bla*TEM	A	*int1*	*papG-III-fimA-bfp*	P8
ASC12	4	AUG-ATM-AMP-S-TOB-CTX-SXT-C-TE	*sul2-sul3-tetA-strA-strB- bla*CTX-3G*-bla*TEM	A	*int1*	*papG-III-fimA-bfp*	P8
ASC13	4	AUG-ATM-AMP-S-TOB-CTX-SXT-C-TE	*sul2-sul3-tetA- strA-strB- bla*CTX-3G*-bla*TEM	A	*int1*	*papG-III-fimA-bfp*	P8
ASC14	4	AUG-ATM-AMP-S-CTX-SXT-C-TE	*sul2-sul3-tetA- strA-strB- bla*CTX-3G*-bla*TEM	A	*int1*	*papG-III-fimA-bfp*	P8
ASC15	4	AUG-ATM-AMP-S-CTX-SXT-C-TE	*sul1-sul2-sul3-tetA- tetB- strA-strB- bla*CTX-3G*- bla*TEM	A	*int1*	*papG-III-fimA-bfp*	P8
ASC16	4	AUG-ATM-AMP-S-TOB-CTX-SXT-C-TE	*sul1-sul3-tetA-cmlA -strA-strB- bla*CTX-3G*-bla*CTX-M9-*bla*TEM	A	*int1*	*papG-III-fimA-bfp*	P8
ASC17	4	AUG-ATM-AMP-S-TOB-CTX-SXT-C-TE	*sul3-tetA-cmlA- strA-strB-blaCTX-3G-bla*CTX-M9*-aadA5-bla*TEM	A	*int1*	*papG-III-fimA-bfp*	P8
ASC19	5	AUG-ATM-AMP-S-TOB-CTX-SXT-C-TE	*sul1-sul3-tetA-cmlA- strA-strB- bla*CTX-M9	B1	*int1*	*papG-III-fimA*	P3
ASC20	5	AUG-ATM-AMP-S-TOB-CTX-SXT-C-TE	*sul3-cmlA- strA-strB-bla*CTX-3G*-bla*CTX-M9*-aadA5-bla*TEM	B1	*int1*	*papG-III*	P2
ASC22	5	AUG-ATM-AMP-S-TOB-CTX-SXT-C-TE	*sul3-cmlA -strA-strB-bla*CTX-M9*-bla*TEM	B1	*int1*	*papG-III*	P2
ASC23	5	AUG-ATM-AMP-S-TOB-CTX-SXT-C-TE	*sul2-sul3-cmlA-strA- bla*CTX-M9*-aadA5-bla*TEM	B1	*-*	*papG-III*	P3
ASC25	5	AUG-ATM-AMP-S-TOB-CTX-SXT-C-TE	*sul2-sul3-tetA-cmlA-bla*CTX*M-strA-strB- bla*TEM	B1	*int1*	*papG-III*	P3
ASC26	5	AUG-ATM-AMP-S-TOB-CTX-SXT-C-TE	*sul1-sul2-sul3-cmlA- strA-strB- bla*CTX-M9*-bla*TEM	B1	*int1*	*papG-III*	P2
ASC27	5	AUG-ATM-AMP-S-TOB-CTX-SXT-C-TE	*sul1-sul2-sul3-tetA-cmlA- strA-strB- bla*CTX-M9*-bla*TEM	B1	*int1*	*papG-III*	P2
ASC28	5	AUG-ATM-AMP-S-TOB-CTX-SXT-C-TE	*sul1-sul3-tetA-cmlA-strA-strB- bla*CTX-M9*-bla*TEM	B1	*int1*	*papG-III*	P2
ASC32	6	ATM-AMP-S-TOB-CTX-CAZ	*sul3- strA-strB-bla*CTX-3G*-bla*CTX-M9	B1	*-*	*papG-III*	P5
ASC33	6	ATM-AMP-S-TOB-CTX-SXT-CAZ	*sul3-strA- bla*CTX-3G*-aadA5-bla*TEM	B1	*-*	*-*	P5
ASC35	13	ATM-AMP-S-CTX-NA-CIP-SXT-C-TE	*qnrS-qnrA-parC-cmlA-tetA- bla*CTX-3G*-bla*CTX-M9*-aac(6)-Ib-aadA5-bla*SHV	D	*int1*	*papG-III*	P1
ASC38	13	ATM-AMP-S-CTX-C-TE	*cmlA-tetA- tetB-bla*CTX-M9*-bla*TEM*-bla*SHV	A	*-*	*papG-III-bfp*	P7
ASC41	13	ATM-AMP-S-CTX-NA-CIP-SXT-TE	*qnrS-qnrA-tetA- bla*CTX-M9 *bla*CTX-3G*-aac(6)-Ib-bla*TEM	A	*int1*	*papG-III-fimA*	P6
ASC42	13	ATM-AMP-S-CTX-NA-CIP-SXT-TE	*qnrA -tetA- tetB-bla*CTX-M9 *bla*CTX-3G*-aac(6)-Ib-bla*TEM	A	*int1*	*papG-III-fimA-bfp*	P7
ASC43	13	ATM-AMP-S-CTX-NA-CIP-SXT-TE	*qnrS-qnrA- tetA-tetB bla*CTX-3G*-aac(6)-Ib-aadA5*	A	*int1*	*papG-III-fimA*	P6
ASC44	13	ATM-AMP-CTX-TE	*tetB-bla*CTX-M9*-bla*CTX-3G	A	*-*	*papG-III-fimA-bfp*	P7
ASC45	13	ATM-AMP-CTX-TE	*tetB-blaCTX-M9-bla*CTX-3G*-bla*TEM	A	*-*	*papG-III-fimA-bfp*	P7
ASC47	13	ATM-AMP-S-TOB-CTX-TE	*tetB-bla*CTX-M9*-bla*CTX-3G	A	*-*	*papG-III-fimA-bfp*	P7

Legend: PFGE—Pulsed-field gel electrophoresis; AUG—amoxicillin–clavulanic acid; ATM—aztreonam; AMP—ampicillin; AK—amikacin; CN—gentamicin; S—streptomycin; TOB—tobramycin; CTX—cefotaxime; CAZ—ceftazidime; NA—nalidixic acid; CIP—ciprofloxacin; SXT—trimethoprim-sulfamethoxazole; C—chloramphenicol; TE—tetracycline.

**Table 2:** Characterization of 19 CTX-resistant *E. coli* isolates that were chosen by PFGE for the analysis of clonal lineages by MLST.

Isolate	Farm	MLST	Resistance Phenotype	Resistance Genotype	Phylogenetic Group	Integrase Gene	Virulence Genes	PFGE Pattern
ASC1	2	ST10	AUG-ATM-AMP-S-TOB-CTX-CAZ-NA-CIP-SXT-C-TE	*sul2-sul3-tetA-cmlA -strA-strB- bla*CTX-3G*-tetB-aac(6)-Ib*	A	*intI*1	*fimA-bfp*	P10
ASC2	3	ST1611	AUG-ATM-AMP-S-TOB-CTX-SXT-C-TE	*sul1-sul3-tetA -strA-strB- blaCTX-3G*	B1	*intI1*	*papG-III-fimA*	P4
ASC3	3	ST8470	AUG-ATM-AMP-S-TOB-CTX-NA-CIP-SXT-C-TE	*sul1-sul3-tetA-qnrS-qnrA-strA-strB- bla*CTX-3G*-aac(6)-Ib-bla*TEM	A	*int1*	*papG-III-fimA-bfp*	P9
ASC5	3	ST1611	AUG-ATM-AMP-CN-S-TOB-CTX-SXT-C-TE	*sul1-sul3-tetA-aac(3)-IV-aac(3)-II- strA-strB- bla*CTX-3G	B1	*-*	*papG-I-fimA II*	P4
ASC9	3	ST8470	AUG-ATM-AMP-S-TOB-CTX-NA-CIP-SXT-C-TE	*sul1-sul3-tetA-qnrS-qnrA- strA-strB- bla*CTX-3G*-tetB-bla*TEM	A	*-*	*fimA-cnf1*	P9
ASC10	4	ST10	AUG-ATM-AMP-S-TOB-CTX-SXT-C-TE	*sul1-sul2-sul3-tetA- strA-strB- bla*CTX-3G*-bla*TEM	A	*int1*	*papG-III-fimA-bfp*	P8
ASC18	5	ST2825	AUG-ATM-AMP-AK-CN-S-TOB-CTX-SXT-C-TE	*sul1-sul2-aac(3)-IV-aac(3)-II-cmlA-strA-strB- bla*CTX-3G*-bla*CTX-M9	B1	*int1*	*papG-III-fimA*	P2
ASC21	5	ST2825	AUG-ATM-AMP-S-TOB-CTX-SXT-C-TE	*sul2-cmlA-strA-strB- blaC*TX-M9-*bla*TEM	B1	*int1*	*papG-III-fimA*	P3
ASC24	5	ST2825	AUG-ATM-AMP-CN-S-TOB-CTX-SXT-C-TE	*sul2-sul3-tetA-aac(3)-IV-aac(3)-II-cmlA- strA-strB-bla*CTX-3G*-bla*CTX-M9*-aadA5-bla*TEM	B1	*int1*	*papG-III*	P3
ASC29	5	ST2825	AUG-ATM-AMP-AK-CN-S-TOB-CTX-SXT-C-TE	*sul1-sul3-tetA-aac(3)-IV-aac(3)-II-cmlA strA-strB-bla*CTX-M9*-bla*TEM	B1	*int1*	*papG-III*	P3
ASC30	6	ST8823	ATM-AMP-S-TOB-CTX-CAZ	*sul3-strB-blaCTX-3G-bla*CTX-M9	B1	*-*	*-*	P5
ASC31	6	ST8823	ATM-AMP-S-TOB-CTX-CAZ	*sul3- strA-strB-bla*CTX-3G	B1	*-*	*papG-III*	P5
ASC34	13	ST457	ATM-AMP-S-CTX-NA-CIP-SXT-C-TE	*tetA-qnrA -qnrS-cmlA- bla*CTX-3G-*aac(6)-Ib-aadA5-bla*TEM	D	*int1*	*papG-III-bfp*	P1
ASC36	13	ST2325	ATM-AMP-CTX-TE	*tetB-bla*CTX-3G*- bla*CTX-M9*-bla*TEM	A	*-*	*papG-III-bfp*	P7
ASC37	13	ST2325	ATM-AMP-S-CTX-CAZ-NA-CIP-SXT-TE	*tetA-qnrS-qnrA-bla*CTX-M9*-bla*SHV	A	*int1*	*papG-III*	P6
ASC39	13	ST2325	ATM-AMP-S-CTX-TE	*tetB-bla*CTX-M9*-aadA5*	A	*-*	*papG-III-bfp*	P6
ASC40	13	ST2325	ATM-AMP-S-CTX-C-TE	*cmlA-tetA-tetB-bla*CTX-M9*-bla*CTX-3G*-aadA5*	A	*-*	*papG-III-bfp*	P6
ASC46	13	ST2325	ATM-AMP-S-TOB-CTX-C-TE	*cmlA-tetA- tetB-bla*CTX-M9	A	*-*	*papG-III-fimA-bfp*	P6
ASC48	13	ST2325	ATM-AMP-S-CTX-C-TE	*cmlA-tetA- bla*CTX-M9*-bla*CTX-3G-*bla*TEM- *bla*SHV	A	*-*	*papG-III-fimA-bfp*	P7

Legend: MLST—Multilocus sequence typing; PFGE—pulsed-field gel electrophoresis; AUG—amoxicillin–clavulanic acid; ATM—aztreonam; AMP—ampicillin; AK—amikacin; CN—gentamicin; S—streptomycin; TOB—tobramycin; CTX—cefotaxime; CAZ—ceftazidime; NA—nalidixic acid; CIP—ciprofloxacin; SXT—trimethoprim-sulfamethoxazole; C—chloramphenicol; TE—tetracycline.

## Data Availability

No new data were created or analyzed in this study. Data sharing is not applicable to this article.
